# 2-[(*E*)-4-Meth­oxy­benzyl­idene]-1,2,3,4-tetra­hydro­naphthalen-1-one

**DOI:** 10.1107/S160053681202805X

**Published:** 2012-06-23

**Authors:** Abdullah M. Asiri, Hassan M. Faidallah, Mohie E. M. Zayed, Seik Weng Ng, Edward R. T. Tiekink

**Affiliations:** a Chemistry Department, Faculty of Science, King Abdulaziz University, PO Box 80203, Jeddah, Saudi Arabia; b Department of Chemistry, University of Malaya, 50603 Kuala Lumpur, Malaysia

## Abstract

Two independent mol­ecules (*A* and *B*) comprise the asymmetric unit of the title compound, C_18_H_16_O_2_. Mol­ecule *B* is virtually superimposable upon *A*. Minor differences are noted in the dihedral angles between the terminal benzene rings of 56.03 (10) and 54.62 (10)°, and in the orientations of meth­oxy groups with respect to the benzene rings to which they are attached [C—O—C—C torsion angles = 169.11 (19) and −172.37 (18)°]. The cyclo­hexene ring of each fused ring system has a screw-boat conformation. In the crystal, C—H⋯π inter­actions assemble mol­ecules into a supra­molecular array in the *ab* plane.

## Related literature

For the activity of related species developed for the treatment of Chagas disease, see: Vera-DiVaio *et al.* (2009 ▶). For the structure of the 2-meth­oxy derivative, see: Dimmock *et al.* (2002 ▶). For conformational analysis, see: Cremer & Pople (1975 ▶).
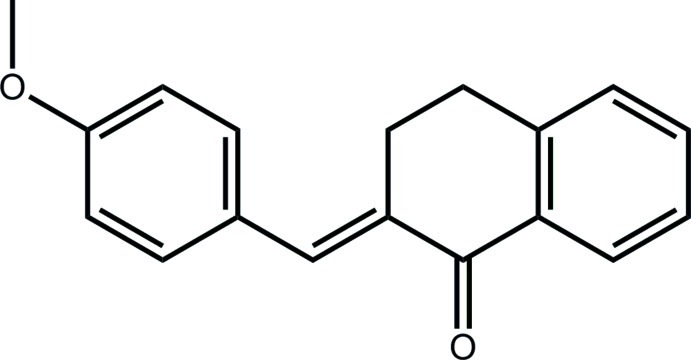


## Experimental

### 

#### Crystal data

C_18_H_16_O_2_
*M* *_r_* = 264.31
Monoclinic, 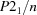

*a* = 6.8289 (4) Å
*b* = 14.7444 (8) Å
*c* = 26.7258 (14) Å
β = 93.757 (5)°
*V* = 2685.2 (3) Å^3^
*Z* = 8
Mo *K*α radiation
μ = 0.08 mm^−1^
*T* = 100 K
0.40 × 0.20 × 0.10 mm

#### Data collection

Agilent SuperNova Dual diffractometer with an Atlas detector
Absorption correction: multi-scan (*CrysAlis PRO*; Agilent, 2012 ▶) *T*_min_ = 0.766, *T*_max_ = 1.000
18691 measured reflections
6195 independent reflections
3813 reflections with *I* > 2σ(*I*)
*R*_int_ = 0.054

#### Refinement

*R*[*F* ^2^ > 2σ(*F* ^2^)] = 0.055
*wR*(*F* ^2^) = 0.187
*S* = 0.99
6195 reflections
361 parameters
H-atom parameters constrained
Δρ_max_ = 0.27 e Å^−3^
Δρ_min_ = −0.25 e Å^−3^

### 

Data collection: *CrysAlis PRO* (Agilent, 2012 ▶); cell refinement: *CrysAlis PRO*; data reduction: *CrysAlis PRO*; program(s) used to solve structure: *SHELXS97* (Sheldrick, 2008 ▶); program(s) used to refine structure: *SHELXL97* (Sheldrick, 2008 ▶); molecular graphics: *ORTEP-3* (Farrugia, 1997 ▶) and *DIAMOND* (Brandenburg, 2006 ▶); software used to prepare material for publication: *publCIF* (Westrip, 2010 ▶).

## Supplementary Material

Crystal structure: contains datablock(s) global, I. DOI: 10.1107/S160053681202805X/bt5948sup1.cif

Structure factors: contains datablock(s) I. DOI: 10.1107/S160053681202805X/bt5948Isup2.hkl

Supplementary material file. DOI: 10.1107/S160053681202805X/bt5948Isup3.cml

Additional supplementary materials:  crystallographic information; 3D view; checkCIF report

## Figures and Tables

**Table 1:** Hydrogen-bond geometry (Å, °) *Cg*1–*Cg*3 are the centroids of the C20–C25, C2–C7 and C12–C17 benzene rings, respectively.

*D*—H⋯*A*	*D*—H	H⋯*A*	*D*⋯*A*	*D*—H⋯*A*
C13—H13⋯*Cg*1^i^	0.95	2.70	3.486 (2)	140
C21—H21⋯*Cg*2^ii^	0.95	2.97	3.595 (2)	124
C31—H31⋯*Cg*2^iii^	0.95	2.64	3.411 (2)	139
C36—H36*B*⋯*Cg*3	0.98	2.88	3.592 (2)	131

Symmetry codes: (i) 
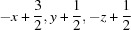
; (ii) 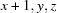; (iii) 
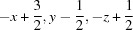
.

**Fig. 1.**
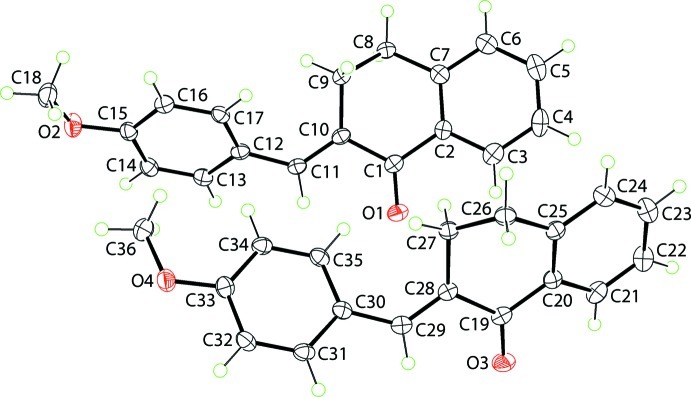
The molecular structure of (I) showing the atom-labelling scheme and displacement ellipsoids at the 50% probability level.

**Fig. 2.**
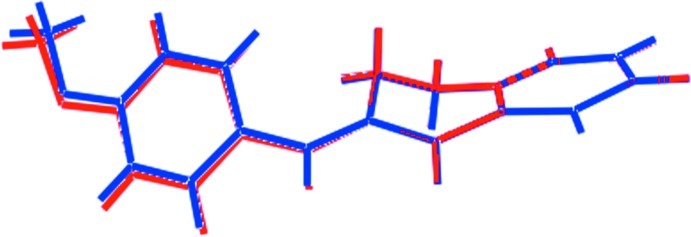
Superimposition of the two independent molecules in (I). The fused ring systems have been superimposed. The O1 and inverted O3-containing molecules are shown as red and blue images, respectively.

**Fig. 3.**
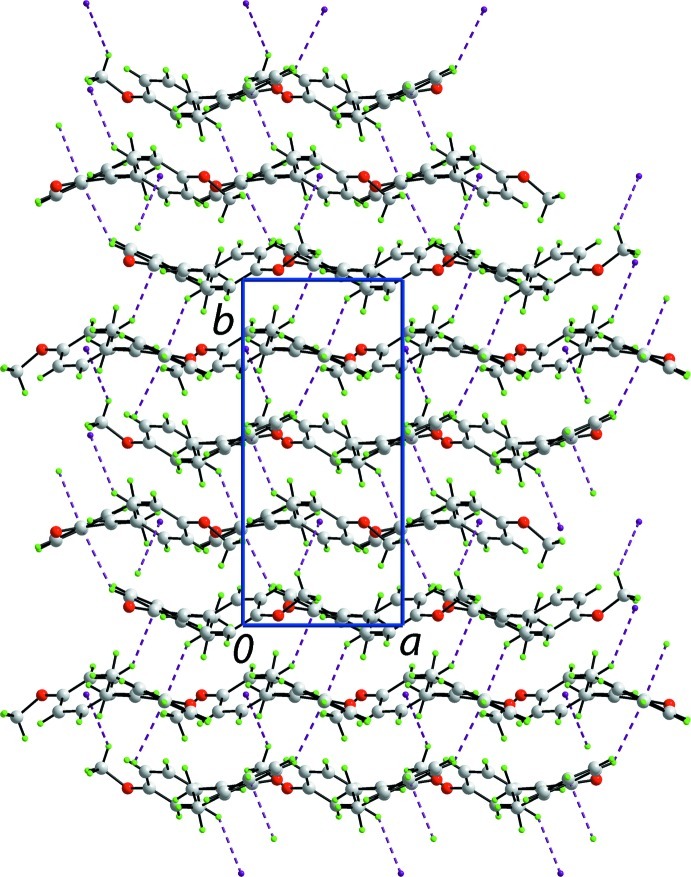
A view of the supramolecular array in the ab plane in (I) mediated by C—H···π interactions shown as purple dashed lines.

**Fig. 4.**
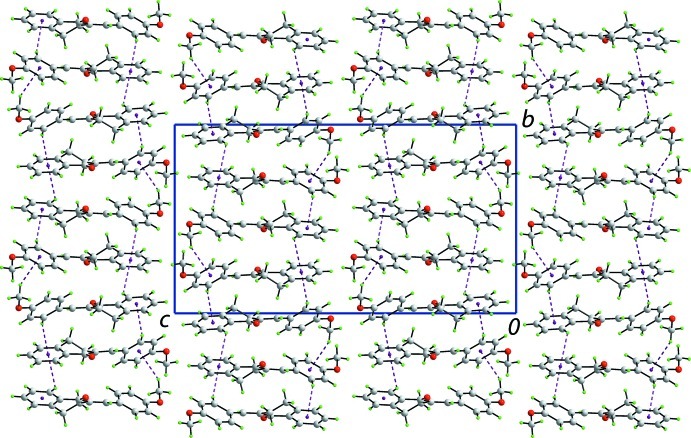
A view in projection down the a axis of the unit-cell contents of (I), showing the stacking supramolecular layers along the c axis. The C—H···π interactions shown as purple dashed lines.

## supplementary crystallographic information

### Comment

The crystal structure of the title compound, 2-(4-methoxybenzylidene)-3,4-dihydro-2*H*-naphthalen-1-one (I), was investigated owing to its relationship to some active compounds developed for the treatment of Chagas disease (Vera-DiVaio *et al.*, 2009).

Two independent molecules comprise the asymmetric unit of (I), Fig. 1. The inverted structure of the O3-containing molecule is virtually super-imposable upon the O1-containing molecule, Fig. 2. The cyclohexene ring of each fused ring system has a screw boat conformation (Cremer & Pople, 1975). A difference between the molecules is seen in the dihedral angles between the terminal benzene rings of 56.03 (10) and 54.62 (10)°, respectively. The methoxy groups are co-planar with the benzene rings to which they are attached as seen in the C18—O2—C15—C14 and C36—O4—C33—C32 torsion angles of 169.11 (19) and -172.37 (18)°, respectively. The conformation about each ethylene bond is *E*. The overall molecular conformation observed for the independent molecules of (I) resembles that seen in the 2-methoxy derivative (Dimmock *et al.*, 2002).

The presence of C—H···π interactions link molecules into a supramolecular array in the *ab* plane in the crystal structure of (I), Fig. 3 and Table 1. Layers stack along the *c* axis with no specific interactions between them (Fig. 4).

### Experimental

A solution of the 4-methoxybenzaldehyde (1.3 g, 0.01 *M*) in ethanol (20 ml) was added to a stirred solution of 1-tetralone (1.46 g,0.01 *M*) in ethanolic KOH (20 ml, 20%), and stirring was maintained at room temperature for 6 h. The reaction mixture was then poured onto water (200 ml) and set aside overnight. The precipitated solid product was collected by filtration, washed with water, dried and recrystallized from ethanol. Yield: 92%.

### Refinement

H-atoms were placed in calculated positions [C—H = 0.95 Å, *U*_iso_(H) = 1.2*U*_eq_(C)] and were included in the refinement in the riding model approximation. One reflection, *i.e*. (-3 6 3), was omitted from the final refinement owing to poor agreement.

### Crystal data

**Table:**

C_18_H_16_O_2_	*F*(000) = 1120
*M**_r_* = 264.31	*D*_x_ = 1.308 Mg m^−^^3^
Monoclinic, *P*2_1_/*n*	Mo *K*α radiation, λ = 0.71073 Å
Hall symbol: -P 2yn	Cell parameters from 3418 reflections
*a* = 6.8289 (4) Å	θ = 2.7–27.5°
*b* = 14.7444 (8) Å	µ = 0.08 mm^−^^1^
*c* = 26.7258 (14) Å	*T* = 100 K
β = 93.757 (5)°	Prism, light-yellow
*V* = 2685.2 (3) Å^3^	0.40 × 0.20 × 0.10 mm
*Z* = 8	

### Data collection

**Table:**

Agilent SuperNova Dual diffractometer with an Atlas detector	6195 independent reflections
Radiation source: SuperNova (Mo) X-ray Source	3813 reflections with *I* > 2σ(*I*)
Mirror monochromator	*R*_int_ = 0.054
Detector resolution: 10.4041 pixels mm^-1^	θ_max_ = 27.6°, θ_min_ = 2.7°
ω scan	*h* = −8→8
Absorption correction: multi-scan (*CrysAlis PRO*; Agilent, 2012)	*k* = −19→15
*T*_min_ = 0.766, *T*_max_ = 1.000	*l* = −34→27
18691 measured reflections	

### Refinement

**Table:**

Refinement on *F*^2^	Primary atom site location: structure-invariant direct methods
Least-squares matrix: full	Secondary atom site location: difference Fourier map
*R*[*F*^2^ > 2σ(*F*^2^)] = 0.055	Hydrogen site location: inferred from neighbouring sites
*wR*(*F*^2^) = 0.187	H-atom parameters constrained
*S* = 0.99	*w* = 1/[σ^2^(*F*_o_^2^) + (0.1*P*)^2^] where *P* = (*F*_o_^2^ + 2*F*_c_^2^)/3
6195 reflections	(Δ/σ)_max_ < 0.001
361 parameters	Δρ_max_ = 0.27 e Å^−^^3^
0 restraints	Δρ_min_ = −0.25 e Å^−^^3^

### Special details

**Table:**

Geometry. All e.s.d.'s (except the e.s.d. in the dihedral angle between two l.s. planes) are estimated using the full covariance matrix. The cell e.s.d.'s are taken into account individually in the estimation of e.s.d.'s in distances, angles and torsion angles; correlations between e.s.d.'s in cell parameters are only used when they are defined by crystal symmetry. An approximate (isotropic) treatment of cell e.s.d.'s is used for estimating e.s.d.'s involving l.s. planes.
Refinement. Refinement of *F*^2^ against ALL reflections. The weighted *R*-factor *wR* and goodness of fit *S* are based on *F*^2^, conventional *R*-factors *R* are based on *F*, with *F* set to zero for negative *F*^2^. The threshold expression of *F*^2^ > σ(*F*^2^) is used only for calculating *R*-factors(gt) *etc*. and is not relevant to the choice of reflections for refinement. *R*-factors based on *F*^2^ are statistically about twice as large as those based on *F*, and *R*- factors based on ALL data will be even larger.

### Fractional atomic coordinates and isotropic or equivalent isotropic displacement parameters (Å^2^)

**Table:**

	*x*	*y*	*z*	*U*_iso_*/*U*_eq_	
O1	0.6701 (2)	0.77015 (11)	0.24286 (5)	0.0275 (4)	
O2	−0.2580 (2)	0.79768 (10)	0.02451 (5)	0.0278 (4)	
O3	1.2130 (2)	0.55355 (11)	0.26086 (6)	0.0292 (4)	
O4	0.2783 (2)	0.52827 (10)	0.04328 (5)	0.0268 (4)	
C1	0.5156 (3)	0.78348 (13)	0.26276 (8)	0.0191 (5)	
C2	0.5128 (3)	0.78434 (13)	0.31863 (7)	0.0192 (5)	
C3	0.6731 (3)	0.74880 (14)	0.34768 (8)	0.0255 (5)	
H3	0.7842	0.7269	0.3318	0.031*	
C4	0.6701 (4)	0.74553 (15)	0.39950 (8)	0.0293 (5)	
H4	0.7778	0.7204	0.4191	0.035*	
C5	0.5088 (4)	0.77917 (15)	0.42253 (8)	0.0289 (5)	
H5	0.5069	0.7775	0.4580	0.035*	
C6	0.3502 (3)	0.81528 (14)	0.39402 (8)	0.0246 (5)	
H6	0.2412	0.8386	0.4103	0.029*	
C7	0.3488 (3)	0.81776 (13)	0.34180 (8)	0.0205 (5)	
C8	0.1784 (3)	0.85520 (14)	0.30966 (7)	0.0207 (5)	
H8A	0.2059	0.9189	0.3008	0.025*	
H8B	0.0590	0.8545	0.3288	0.025*	
C9	0.1421 (3)	0.79936 (14)	0.26173 (8)	0.0203 (5)	
H9A	0.1016	0.7372	0.2705	0.024*	
H9B	0.0343	0.8271	0.2404	0.024*	
C10	0.3253 (3)	0.79488 (13)	0.23293 (8)	0.0186 (4)	
C11	0.3302 (3)	0.80015 (13)	0.18286 (7)	0.0188 (5)	
H11	0.4575	0.8033	0.1706	0.023*	
C12	0.1669 (3)	0.80180 (13)	0.14431 (7)	0.0191 (5)	
C13	0.1890 (3)	0.84882 (14)	0.09931 (7)	0.0208 (5)	
H13	0.3059	0.8822	0.0953	0.025*	
C14	0.0440 (3)	0.84745 (14)	0.06078 (8)	0.0216 (5)	
H14	0.0606	0.8809	0.0310	0.026*	
C15	−0.1261 (3)	0.79728 (14)	0.06547 (7)	0.0209 (5)	
C16	−0.1524 (3)	0.74988 (14)	0.10958 (7)	0.0205 (5)	
H16	−0.2689	0.7159	0.1131	0.025*	
C17	−0.0066 (3)	0.75278 (13)	0.14846 (7)	0.0192 (5)	
H17	−0.0254	0.7206	0.1786	0.023*	
C18	−0.4166 (3)	0.73482 (16)	0.02424 (9)	0.0307 (5)	
H18A	−0.5007	0.7424	−0.0066	0.046*	
H18B	−0.4935	0.7460	0.0533	0.046*	
H18C	−0.3647	0.6728	0.0258	0.046*	
C19	1.0573 (3)	0.54246 (13)	0.28040 (8)	0.0193 (5)	
C20	1.0499 (3)	0.54485 (13)	0.33611 (8)	0.0191 (5)	
C21	1.2083 (3)	0.58197 (14)	0.36510 (8)	0.0245 (5)	
H21	1.3201	0.6033	0.3493	0.029*	
C22	1.2022 (3)	0.58761 (14)	0.41678 (8)	0.0274 (5)	
H22	1.3095	0.6130	0.4365	0.033*	
C23	1.0381 (3)	0.55593 (14)	0.43966 (8)	0.0268 (5)	
H23	1.0329	0.5605	0.4750	0.032*	
C24	0.8820 (3)	0.51760 (14)	0.41117 (8)	0.0234 (5)	
H24	0.7722	0.4949	0.4273	0.028*	
C25	0.8846 (3)	0.51211 (14)	0.35905 (8)	0.0199 (5)	
C26	0.7168 (3)	0.47224 (14)	0.32696 (8)	0.0225 (5)	
H26A	0.5962	0.4737	0.3456	0.027*	
H26B	0.7462	0.4081	0.3194	0.027*	
C27	0.6822 (3)	0.52514 (14)	0.27784 (7)	0.0201 (5)	
H27A	0.5783	0.4948	0.2564	0.024*	
H27B	0.6365	0.5871	0.2852	0.024*	
C28	0.8670 (3)	0.53087 (13)	0.25012 (7)	0.0184 (5)	
C29	0.8732 (3)	0.52698 (13)	0.19981 (8)	0.0197 (5)	
H29	1.0006	0.5245	0.1876	0.024*	
C30	0.7102 (3)	0.52606 (13)	0.16160 (7)	0.0185 (4)	
C31	0.7301 (3)	0.47902 (14)	0.11619 (8)	0.0214 (5)	
H31	0.8470	0.4458	0.1118	0.026*	
C32	0.5845 (3)	0.48000 (14)	0.07810 (8)	0.0221 (5)	
H32	0.6005	0.4471	0.0481	0.027*	
C33	0.4132 (3)	0.52956 (14)	0.08374 (8)	0.0214 (5)	
C34	0.3894 (3)	0.57657 (14)	0.12784 (7)	0.0207 (5)	
H34	0.2727	0.6102	0.1317	0.025*	
C35	0.5359 (3)	0.57460 (14)	0.16638 (7)	0.0203 (5)	
H35	0.5176	0.6067	0.1965	0.024*	
C36	0.1135 (3)	0.58699 (15)	0.04526 (8)	0.0268 (5)	
H36A	0.0275	0.5796	0.0147	0.040*	
H36B	0.1586	0.6500	0.0479	0.040*	
H36C	0.0408	0.5718	0.0746	0.040*	

### Atomic displacement parameters (Å^2^)

**Table:**

	*U*^11^	*U*^22^	*U*^33^	*U*^12^	*U*^13^	*U*^23^
O1	0.0184 (8)	0.0352 (9)	0.0293 (9)	0.0018 (7)	0.0038 (7)	−0.0026 (7)
O2	0.0298 (9)	0.0303 (9)	0.0224 (8)	−0.0040 (7)	−0.0051 (7)	0.0049 (6)
O3	0.0190 (8)	0.0375 (10)	0.0316 (9)	−0.0025 (7)	0.0057 (7)	−0.0020 (7)
O4	0.0313 (9)	0.0285 (9)	0.0200 (8)	0.0075 (7)	−0.0034 (7)	−0.0018 (6)
C1	0.0190 (11)	0.0134 (10)	0.0248 (11)	0.0002 (8)	0.0009 (9)	−0.0007 (8)
C2	0.0211 (11)	0.0151 (10)	0.0209 (11)	−0.0019 (8)	−0.0016 (9)	0.0001 (8)
C3	0.0256 (12)	0.0184 (11)	0.0320 (13)	−0.0009 (9)	−0.0017 (10)	−0.0032 (9)
C4	0.0392 (14)	0.0192 (12)	0.0275 (12)	−0.0014 (10)	−0.0125 (11)	0.0008 (9)
C5	0.0428 (15)	0.0212 (12)	0.0222 (12)	−0.0095 (10)	−0.0032 (11)	0.0017 (9)
C6	0.0301 (13)	0.0182 (11)	0.0258 (12)	−0.0049 (9)	0.0057 (10)	−0.0001 (9)
C7	0.0228 (12)	0.0161 (11)	0.0227 (11)	−0.0065 (8)	0.0022 (9)	−0.0016 (8)
C8	0.0188 (11)	0.0207 (11)	0.0230 (11)	0.0024 (8)	0.0034 (9)	−0.0009 (8)
C9	0.0163 (11)	0.0214 (11)	0.0231 (11)	0.0001 (8)	0.0007 (9)	−0.0002 (8)
C10	0.0163 (11)	0.0155 (10)	0.0240 (11)	0.0015 (8)	0.0019 (9)	−0.0009 (8)
C11	0.0163 (11)	0.0173 (11)	0.0231 (11)	0.0010 (8)	0.0031 (9)	−0.0018 (8)
C12	0.0186 (11)	0.0162 (11)	0.0227 (11)	0.0024 (8)	0.0035 (9)	−0.0023 (8)
C13	0.0229 (12)	0.0174 (11)	0.0226 (11)	−0.0006 (9)	0.0044 (9)	−0.0019 (8)
C14	0.0273 (12)	0.0191 (11)	0.0185 (11)	0.0019 (9)	0.0028 (9)	0.0023 (8)
C15	0.0239 (12)	0.0201 (11)	0.0186 (11)	0.0036 (9)	0.0006 (9)	−0.0010 (8)
C16	0.0204 (11)	0.0176 (11)	0.0237 (12)	−0.0002 (8)	0.0024 (9)	−0.0017 (8)
C17	0.0229 (11)	0.0176 (11)	0.0175 (11)	0.0028 (8)	0.0039 (9)	0.0003 (8)
C18	0.0262 (13)	0.0367 (14)	0.0280 (13)	−0.0040 (10)	−0.0058 (10)	0.0001 (10)
C19	0.0167 (11)	0.0141 (10)	0.0270 (12)	−0.0012 (8)	0.0019 (9)	−0.0009 (8)
C20	0.0211 (11)	0.0144 (10)	0.0218 (11)	0.0017 (8)	0.0013 (9)	0.0021 (8)
C21	0.0209 (12)	0.0193 (12)	0.0328 (13)	−0.0026 (9)	−0.0023 (10)	0.0012 (9)
C22	0.0332 (13)	0.0192 (12)	0.0288 (13)	−0.0052 (10)	−0.0054 (10)	0.0012 (9)
C23	0.0388 (14)	0.0186 (12)	0.0226 (12)	−0.0022 (10)	−0.0003 (10)	−0.0003 (9)
C24	0.0284 (12)	0.0177 (11)	0.0244 (12)	−0.0012 (9)	0.0043 (10)	0.0001 (8)
C25	0.0211 (11)	0.0158 (10)	0.0228 (11)	0.0032 (8)	0.0011 (9)	0.0009 (8)
C26	0.0208 (11)	0.0224 (12)	0.0246 (11)	−0.0017 (9)	0.0039 (9)	0.0003 (9)
C27	0.0186 (11)	0.0202 (11)	0.0217 (11)	−0.0009 (9)	0.0028 (9)	0.0003 (8)
C28	0.0171 (11)	0.0148 (10)	0.0233 (11)	−0.0012 (8)	0.0023 (9)	0.0009 (8)
C29	0.0201 (11)	0.0148 (11)	0.0246 (11)	−0.0008 (8)	0.0047 (9)	0.0007 (8)
C30	0.0209 (11)	0.0161 (10)	0.0190 (11)	−0.0016 (8)	0.0048 (9)	0.0028 (8)
C31	0.0233 (12)	0.0190 (11)	0.0226 (11)	0.0045 (9)	0.0070 (9)	0.0035 (8)
C32	0.0299 (12)	0.0197 (11)	0.0169 (11)	0.0030 (9)	0.0040 (9)	−0.0010 (8)
C33	0.0261 (12)	0.0190 (11)	0.0191 (11)	−0.0007 (9)	0.0002 (9)	0.0035 (8)
C34	0.0219 (11)	0.0181 (11)	0.0226 (11)	0.0041 (9)	0.0049 (9)	0.0017 (8)
C35	0.0255 (12)	0.0177 (11)	0.0183 (11)	0.0009 (9)	0.0044 (9)	0.0009 (8)
C36	0.0251 (12)	0.0270 (12)	0.0279 (12)	0.0031 (10)	−0.0026 (10)	0.0032 (9)

### Geometric parameters (Å, º)

**Table:**

O1—C1	1.229 (2)	C18—H18A	0.9800
O2—C15	1.371 (2)	C18—H18B	0.9800
O2—C18	1.426 (3)	C18—H18C	0.9800
O3—C19	1.226 (2)	C19—C20	1.494 (3)
O4—C33	1.374 (2)	C19—C28	1.495 (3)
O4—C36	1.424 (2)	C20—C21	1.400 (3)
C1—C10	1.489 (3)	C20—C25	1.405 (3)
C1—C2	1.494 (3)	C21—C22	1.387 (3)
C2—C3	1.401 (3)	C21—H21	0.9500
C2—C7	1.404 (3)	C22—C23	1.392 (3)
C3—C4	1.387 (3)	C22—H22	0.9500
C3—H3	0.9500	C23—C24	1.389 (3)
C4—C5	1.388 (3)	C23—H23	0.9500
C4—H4	0.9500	C24—C25	1.396 (3)
C5—C6	1.389 (3)	C24—H24	0.9500
C5—H5	0.9500	C25—C26	1.505 (3)
C6—C7	1.395 (3)	C26—C27	1.532 (3)
C6—H6	0.9500	C26—H26A	0.9900
C7—C8	1.506 (3)	C26—H26B	0.9900
C8—C9	1.529 (3)	C27—C28	1.507 (3)
C8—H8A	0.9900	C27—H27A	0.9900
C8—H8B	0.9900	C27—H27B	0.9900
C9—C10	1.512 (3)	C28—C29	1.349 (3)
C9—H9A	0.9900	C29—C30	1.460 (3)
C9—H9B	0.9900	C29—H29	0.9500
C10—C11	1.343 (3)	C30—C35	1.402 (3)
C11—C12	1.468 (3)	C30—C31	1.412 (3)
C11—H11	0.9500	C31—C32	1.375 (3)
C12—C17	1.398 (3)	C31—H31	0.9500
C12—C13	1.405 (3)	C32—C33	1.396 (3)
C13—C14	1.381 (3)	C32—H32	0.9500
C13—H13	0.9500	C33—C34	1.386 (3)
C14—C15	1.389 (3)	C34—C35	1.388 (3)
C14—H14	0.9500	C34—H34	0.9500
C15—C16	1.392 (3)	C35—H35	0.9500
C16—C17	1.392 (3)	C36—H36A	0.9800
C16—H16	0.9500	C36—H36B	0.9800
C17—H17	0.9500	C36—H36C	0.9800
			
C15—O2—C18	117.43 (16)	H18B—C18—H18C	109.5
C33—O4—C36	116.96 (16)	O3—C19—C20	120.56 (19)
O1—C1—C10	122.05 (18)	O3—C19—C28	122.16 (19)
O1—C1—C2	120.08 (19)	C20—C19—C28	117.21 (17)
C10—C1—C2	117.80 (17)	C21—C20—C25	120.44 (19)
C3—C2—C7	120.22 (19)	C21—C20—C19	119.04 (18)
C3—C2—C1	119.34 (18)	C25—C20—C19	120.51 (18)
C7—C2—C1	120.41 (18)	C22—C21—C20	120.1 (2)
C4—C3—C2	120.3 (2)	C22—C21—H21	119.9
C4—C3—H3	119.9	C20—C21—H21	119.9
C2—C3—H3	119.9	C21—C22—C23	119.7 (2)
C5—C4—C3	119.6 (2)	C21—C22—H22	120.2
C5—C4—H4	120.2	C23—C22—H22	120.2
C3—C4—H4	120.2	C24—C23—C22	120.5 (2)
C4—C5—C6	120.5 (2)	C24—C23—H23	119.8
C4—C5—H5	119.8	C22—C23—H23	119.8
C6—C5—H5	119.8	C23—C24—C25	120.7 (2)
C5—C6—C7	120.8 (2)	C23—C24—H24	119.6
C5—C6—H6	119.6	C25—C24—H24	119.6
C7—C6—H6	119.6	C24—C25—C20	118.59 (19)
C6—C7—C2	118.61 (19)	C24—C25—C26	122.15 (18)
C6—C7—C8	122.30 (18)	C20—C25—C26	119.27 (18)
C2—C7—C8	119.09 (18)	C25—C26—C27	111.00 (17)
C7—C8—C9	110.93 (17)	C25—C26—H26A	109.4
C7—C8—H8A	109.5	C27—C26—H26A	109.4
C9—C8—H8A	109.5	C25—C26—H26B	109.4
C7—C8—H8B	109.5	C27—C26—H26B	109.4
C9—C8—H8B	109.5	H26A—C26—H26B	108.0
H8A—C8—H8B	108.0	C28—C27—C26	111.27 (17)
C10—C9—C8	110.85 (17)	C28—C27—H27A	109.4
C10—C9—H9A	109.5	C26—C27—H27A	109.4
C8—C9—H9A	109.5	C28—C27—H27B	109.4
C10—C9—H9B	109.5	C26—C27—H27B	109.4
C8—C9—H9B	109.5	H27A—C27—H27B	108.0
H9A—C9—H9B	108.1	C29—C28—C19	117.47 (18)
C11—C10—C1	117.53 (18)	C29—C28—C27	124.72 (19)
C11—C10—C9	125.45 (19)	C19—C28—C27	117.81 (17)
C1—C10—C9	117.02 (17)	C28—C29—C30	128.69 (19)
C10—C11—C12	129.27 (19)	C28—C29—H29	115.7
C10—C11—H11	115.4	C30—C29—H29	115.7
C12—C11—H11	115.4	C35—C30—C31	117.31 (19)
C17—C12—C13	117.51 (19)	C35—C30—C29	122.98 (18)
C17—C12—C11	122.97 (18)	C31—C30—C29	119.60 (18)
C13—C12—C11	119.34 (18)	C32—C31—C30	121.70 (19)
C14—C13—C12	121.27 (19)	C32—C31—H31	119.1
C14—C13—H13	119.4	C30—C31—H31	119.1
C12—C13—H13	119.4	C31—C32—C33	119.71 (19)
C13—C14—C15	120.21 (19)	C31—C32—H32	120.1
C13—C14—H14	119.9	C33—C32—H32	120.1
C15—C14—H14	119.9	O4—C33—C34	124.49 (19)
O2—C15—C14	115.78 (18)	O4—C33—C32	115.54 (18)
O2—C15—C16	124.29 (19)	C34—C33—C32	120.0 (2)
C14—C15—C16	119.9 (2)	C33—C34—C35	120.11 (19)
C15—C16—C17	119.39 (19)	C33—C34—H34	119.9
C15—C16—H16	120.3	C35—C34—H34	119.9
C17—C16—H16	120.3	C34—C35—C30	121.19 (19)
C16—C17—C12	121.67 (19)	C34—C35—H35	119.4
C16—C17—H17	119.2	C30—C35—H35	119.4
C12—C17—H17	119.2	O4—C36—H36A	109.5
O2—C18—H18A	109.5	O4—C36—H36B	109.5
O2—C18—H18B	109.5	H36A—C36—H36B	109.5
H18A—C18—H18B	109.5	O4—C36—H36C	109.5
O2—C18—H18C	109.5	H36A—C36—H36C	109.5
H18A—C18—H18C	109.5	H36B—C36—H36C	109.5
			
O1—C1—C2—C3	−15.4 (3)	O3—C19—C20—C21	16.0 (3)
C10—C1—C2—C3	161.88 (18)	C28—C19—C20—C21	−160.95 (18)
O1—C1—C2—C7	166.67 (19)	O3—C19—C20—C25	−165.3 (2)
C10—C1—C2—C7	−16.1 (3)	C28—C19—C20—C25	17.7 (3)
C7—C2—C3—C4	0.6 (3)	C25—C20—C21—C22	−0.7 (3)
C1—C2—C3—C4	−177.35 (18)	C19—C20—C21—C22	177.95 (19)
C2—C3—C4—C5	−1.1 (3)	C20—C21—C22—C23	0.2 (3)
C3—C4—C5—C6	0.6 (3)	C21—C22—C23—C24	0.9 (3)
C4—C5—C6—C7	0.6 (3)	C22—C23—C24—C25	−1.5 (3)
C5—C6—C7—C2	−1.1 (3)	C23—C24—C25—C20	1.0 (3)
C5—C6—C7—C8	179.15 (19)	C23—C24—C25—C26	−179.12 (19)
C3—C2—C7—C6	0.5 (3)	C21—C20—C25—C24	0.1 (3)
C1—C2—C7—C6	178.42 (18)	C19—C20—C25—C24	−178.53 (18)
C3—C2—C7—C8	−179.74 (18)	C21—C20—C25—C26	−179.76 (18)
C1—C2—C7—C8	−1.8 (3)	C19—C20—C25—C26	1.6 (3)
C6—C7—C8—C9	−142.08 (19)	C24—C25—C26—C27	142.0 (2)
C2—C7—C8—C9	38.1 (2)	C20—C25—C26—C27	−38.1 (3)
C7—C8—C9—C10	−56.1 (2)	C25—C26—C27—C28	54.7 (2)
O1—C1—C10—C11	−7.2 (3)	O3—C19—C28—C29	4.3 (3)
C2—C1—C10—C11	175.64 (17)	C20—C19—C28—C29	−178.81 (17)
O1—C1—C10—C9	172.68 (19)	O3—C19—C28—C27	−175.23 (19)
C2—C1—C10—C9	−4.5 (3)	C20—C19—C28—C27	1.7 (3)
C8—C9—C10—C11	−140.1 (2)	C26—C27—C28—C29	143.1 (2)
C8—C9—C10—C1	40.1 (2)	C26—C27—C28—C19	−37.4 (2)
C1—C10—C11—C12	172.93 (19)	C19—C28—C29—C30	−172.60 (19)
C9—C10—C11—C12	−6.9 (3)	C27—C28—C29—C30	6.9 (3)
C10—C11—C12—C17	−35.3 (3)	C28—C29—C30—C35	35.2 (3)
C10—C11—C12—C13	149.7 (2)	C28—C29—C30—C31	−148.7 (2)
C17—C12—C13—C14	0.6 (3)	C35—C30—C31—C32	−0.4 (3)
C11—C12—C13—C14	175.92 (18)	C29—C30—C31—C32	−176.69 (18)
C12—C13—C14—C15	−1.5 (3)	C30—C31—C32—C33	0.9 (3)
C18—O2—C15—C14	169.11 (19)	C36—O4—C33—C34	6.9 (3)
C18—O2—C15—C16	−10.1 (3)	C36—O4—C33—C32	−172.37 (18)
C13—C14—C15—O2	−177.76 (18)	C31—C32—C33—O4	178.49 (17)
C13—C14—C15—C16	1.5 (3)	C31—C32—C33—C34	−0.8 (3)
O2—C15—C16—C17	178.62 (18)	O4—C33—C34—C35	−179.09 (18)
C14—C15—C16—C17	−0.5 (3)	C32—C33—C34—C35	0.1 (3)
C15—C16—C17—C12	−0.4 (3)	C33—C34—C35—C30	0.5 (3)
C13—C12—C17—C16	0.3 (3)	C31—C30—C35—C34	−0.3 (3)
C11—C12—C17—C16	−174.79 (18)	C29—C30—C35—C34	175.87 (18)

### Hydrogen-bond geometry (Å, º)

Cg1–Cg3 are the centroids of the C20–C25, C2–C7 and C12–C17 benzene rings, respectively.

**Table:**

*D*—H···*A*	*D*—H	H···*A*	*D*···*A*	*D*—H···*A*
C13—H13···*Cg*1^i^	0.95	2.70	3.486 (2)	140
C21—H21···*Cg*2^ii^	0.95	2.97	3.595 (2)	124
C31—H31···*Cg*2^iii^	0.95	2.64	3.411 (2)	139
C36—H36*B*···*Cg*3	0.98	2.88	3.592 (2)	131

Symmetry codes: (i) −*x*+3/2, *y*+1/2, −*z*+1/2; (ii) *x*+1, *y*, *z*; (iii) −*x*+3/2, *y*−1/2, −*z*+1/2.
